# A Closer Look into the Role of Protein Tau in the Identification of Promising Therapeutic Targets for Alzheimer’s Disease

**DOI:** 10.3390/brainsci8090162

**Published:** 2018-08-26

**Authors:** Rubayat Islam Khan, Saif Shahriar Rahman Nirzhor, Barnaly Rashid

**Affiliations:** 1 Department of Pharmacy, BRAC University, Dhaka 1212, Bangladesh; rubayat.khan@bracu.ac.bd (R.I.K.); saif.rahman@bracu.ac.bd (S.S.R.N.); 2 Harvard Medical School, Harvard University, Boston, MA 02114, USA

**Keywords:** protein tau, Alzheimer’s disease, neurodegenerative disease, synaptic dysfunction, Aβ-peptides, tau-imaging

## Abstract

One of the most commonly known chronic neurodegenerative disorders, Alzheimer’s disease (AD), manifests the common type of dementia in 60–80% of cases. From a clinical standpoint, a patent cognitive decline and a severe change in personality, as caused by a loss of neurons, is usually evident in AD with about 50 million people affected in 2016. The disease progression in patients is distinguished by a gradual plummet in cognitive functions, eliciting symptoms such as memory loss, and eventually requiring full-time medical care. From a histopathological standpoint, the defining characteristics are intracellular aggregations of hyper-phosphorylated tau protein, known as neurofibrillary tangles (NFT), and depositions of amyloid β-peptides (Aβ) in the brain. The abnormal phosphorylation of tau protein is attributed to a wide gamut of neurological disorders known as tauopathies. In addition to the hyperphosphorylated tau lesions, neuroinflammatory processes could occur in a sustained manner through astro-glial activation, resulting in the disease progression. Recent findings have suggested a strong interplay between the mechanism of Tau phosphorylation, disruption of microtubules, and synaptic loss and pathology of AD. The mechanisms underlying these interactions along with their respective consequences in Tau pathology are still ill-defined. Thus, in this review: (1) we highlight the interplays existing between Tau pathology and AD; and (2) take a closer look into its role while identifying some promising therapeutic advances including state of the art imaging techniques.

## 1. Introduction

Originally, tau was recognized as a protein in the cytoplasm and was tagged with the role of stabilizing microtubules. Tau is coded by the microtubule associated protein tau (MAPT) gene and is usually abundant in neuronal cells. It has shown six different isoforms in neuronal cells because of differential splicing and is associated with multi-faceted functions even though some of these functions are not clearly understood [1,2]. This protein has been shown to play a critical role in Alzheimer’s disease (AD) pathology and could be the future in terms of treating AD and engendering new therapeutic targets. Several studies have tried and unfortunately failed to successfully target the Aβ-peptide buildup in the brain. Recent studies indicate that it may as well be the case that the Aβ pathology becomes significant many years after tau aggregations start to form in an AD patient [3,4]. These findings provide impetus for shifting the focus from the Aβ pathology to the role of protein tau in AD, so that effective strategies for treating AD may be identified [4,5,6]. 

**Figure 1:**
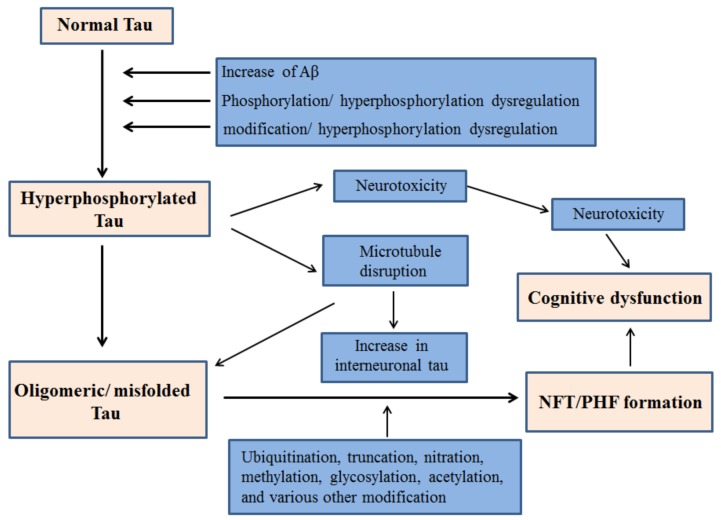
Proposed mechanism of NFT generation leading to cognitive dysfunction by hyperphosphorylated Tau.

Primarily, tau facilitates the assembly of microtubules and the regulation of their stability, thereby eventuating cytoskeleton maintenance, organelle axonal transport and overall neuronal morphology [7,8,9]. In addition, tau is pivotal in stabilizing genomes and protecting DNA integrity [10,11,12]. With normal human aging, the brain becomes vulnerable to neuronal tauopathies and increased accumulation of protein tau in glial cells. Primary age related tauopathy (PART) and aging related tau astrogliopathy (ARTAG) are recently introduced neuropathological entities. The morphological spectrum of tau immunoreactivity as present in glial cells of the aging human brain is described by ARTAG, regardless of the existence of any concurrent neurological disorders [13]. Neurofibrillary Tangles (NFT) are hyperphosphorylated tau protein aggregates most commonly known as a primary marker of Alzheimer's disease. NFT are abundantly present in neurons of old-aged individuals as described by PART, with cognitive changes ranging from normal to amnestic [14]. The majority of Tau proteins are located in the axons while the dendrites consist of a smaller proportion physiologically distributed in them. Even though, their post synaptic function still remains imprecise, tau has been previously implicated in synaptic plasticity. [15,16,17,18,19]. In addition to axons and dendrites, nuclear tau are involved in the regulation of transcriptional activity and DNA/RNA maintenance under various physiological conditions [10,11,20,21]. Recent evidences signify Tau’s role as a signaling molecule in the regulation of the brain insulin pathway where it is implicated in the inhibition of phosphatase and tensin homolog (PTEN) [22,23]. Various etiological factors contribute to the abnormal phosphorylation of tau and subsequent NFT generation and cognitive dysfunction. A schematic of such mechanisms is outlined in Figure 1.

## 2. Tau Hyperphosphorylation in AD

The miscellaneous attributes and interactions of Tau with its protein analogues are governed by phosphorylation, ubiquitination, truncation, nitration, methylation, glycosylation, acetylation and various other post-translational modifications (PTMs) [24,25]. The microtubule binding region and proline-rich domain of Tau pertain 85 putative phosphorylation sites [26,27]. These phosphorylation sites are identified through the use of mass spectroscopy or phosphor-specific antibodies [28,29]. Phosphorylation states in tauopathies are governed by a number of serine/threonine/tyrosine kinases as well as phosphatases [28]. The intriguing process of tau phosphorylation in AD comprises of tau phosphorylation early in the pathogenesis, formation of the epitopes, initiation of structural changes that promote the activity of secondary kinases; thus following a hierarchical process. A number of studies have demonstrated that the epitopes detected by the antibody AT100 and recognizing paired-helical filaments (PHF) are attributed to the sequential phosphorylation by GSK3β and PKA at Ser214 and Thr212, in addition to Thr 205, Ser202, Ser199 and Thr205 phosphorylation [30,31]. Immune cells can recognize the epitopes generated by Tau phosphorylation. The activation of Tau is enhanced by the expression of Tau by microglial cells [27]. However, the detailed processes resulting in Tau phosphorylation still remain to be explored but accordingly, structural changes promote its detachment from microtubules thereby, producing soluble free tau in high quantities. This gives rise to different degrees of neurotoxicity as Tau hyperphosphorylation favors a gradual self-assembly of Tau, transforming into oligomeric forms and PHF through the disease progression [28].

The enhancement of tau phosphorylation arises from the activity of a number of tyrosine (Tyr) kinases and some serine/threonine (Ser/Thr) kinases. The casein kinases, Ser/Thr kinases GSK-3β, and cyclin-dependent kinase 5 (cdk5) phosphorylate tau in AD and are instrumental in the progression of the disease. Researchers have also regarded them as efficient therapeutic targets that hold significant promises against tau-induced toxicity [28,32]. In general, Tau is phosphorylated at a greater number of sites by proline-directed kinases as compared to phosphorylation by kinases that are not directed by proline. However, such kinases (e.g., PKA/calcium/calmodulin kinase II) phosphorylates tau at very few sites but they facilitate the progressive tau phosphorylation by kinases that are proline-directed, namely GSK-3β and cdk5 [33,34]. Abnormal tau phosphorylation is a key player in AD progression and pathogenesis. In different brain regions, the phosphorylation patterns of numerous proteins are altered synergistically; thus transitioning to a symptomatic state of the disease. A large number of abnormally phosphorylated Tau are crucial in synaptic function and cytoskeletal maintenance [35]. In addition to phosphorylation of tau at 42 residues, GSK-3β regulates various other cellular processes and is a key player in the pathogenesis of AD [28]. Table 1 highlights some major enzymes that cause tau phosphorylation at various Ser/Thr sites. In various animal models, GSK-3β has shown to stimulate phosphorylation of tau in neuronal cell cultures, promote the formation of tangle-like filaments, eventuate tau hyperphosphorylation, resulting in cognitive decline [36,37,38]. Presenilin 1—a γ-secretase complex modulates the regulation of tau phosphorylation mediated by GSK-3β. Presenilin 1 also depicted enhanced ability to bind and stimulate tau-directed kinase activity by GSK-3β in AD-related mutations [39]. In diverse neurodegenerative conditions including AD, GSK-3β facilitates cell apoptosis. This is facilitated by the proapoptotic stimuli that affect the distribution of GSK-3β within the cells, thereby initiating the cell death signaling networks. Studies conducted in SH-SY5Y cell lines (human neuroblastoma cells)have shown that GSK-3β is localized primarily in the cytosol, however the post-apoptotic intercession facilitates its aggregation in the nucleus where it interacts with nuclear substrates [40]. NFT-tau pathogenesis in AD progresses in a spatio-temporal manner [3,9,41,42]. This is strikingly different from the process of the deposition of Aβ plaque where the pattern of localization and quantity is of little significance in the pathogenesis of AD, and leads to gradual cognitive decline [43,44]. The loss of neurons is more profound as compared to NFT formation in the AD brain [44].

### 2.1. GSK-3β Inhibition in Tau

Tau hyperphosphorylation and NFT formation is a direct outcome of GSK-3β mediated cognitive decline. The first sign of the disease is identified by the moderate somatodendritic accumulation of nonfibrillar tau that is conformationally altered [61]. Though it is well established that tau’s function in the stabilization of microtubules is attenuated by its hyperphosphorylation, however its constructive part in tau aggregation still remains ill-defined. Studies have previously emphasized that hyperphosphorylation has a positive correlation with PHF formation, however recent investigations have suggested that just hyperphosphorylation is not sufficient enough for the formation of fibrils, although increased phosphorylation promotes oligomer formation [62,63]. Apolipoprotein E (ApoE) is a class of proteins with implications in lipid metabolism in the body with attributed importance in AD. In addition to influencing the accumulation and removal of Aβ, isoforms of ApoE can condition tau and microtubule through modulation of signal transduction pathways that are responsible for tau kinase activity [64]. In a study conducted by Hoe et al., the treatment of primary neurons with three different ApoE isoforms showed decreased aggregation of phosphorylated tau, increased levels of unphosphorylated tau, inhibited phosphorylation of GSK-3β and altered the localization pattern of tau in neuronal cells through extracellular interactions [64]. ApoE isoforms might also bind tau specifically and inhibit tau phosphorylation. GSK-3 mediated tau phosphorylation is increased by isoform ApoE4 due to less specific binding of tau [65]. In addition, truncated forms of ApoE (present in the AD brain) facilitate the generation of inclusions that are NFT-like and comprise of high molecular weight phosphorylated neurofilaments and also phosphorylated tau [66]. The expression and activity of protein phosphatases 1,2A,2B and 5 (PP1, PP2A, PP2B, PP5) are altered in the AD brain [28,67]. Phosphoprotein phosphatases PP1, PP2A, PP2B, and PP5 dephosphorylates tau at variegated sites with PP2A being the key player in tau dephosphorylation with downregulated activity in the AD brain [32,67,68]. GSK-3β activation gives rise to increased accumulation of the inhibitor-2 of protein phosphatase-2A (I_2_^PP2A^) and thereby decreases the activity of PP2A. The increase in I_2_^PP2A^ inhibits PP2A activity and thereby hyperphosphorylates tau. Conversely, the downregulation of I_2_^PP2A^ reinstates the activity of PP2A and attenuates the accumulation and phosphorylation of tau, inhibits GSK-3β through the activation of PKA, improves cognitive functions, and dendritic plasticity in studies conducted with human tau transgenic mice. Thus, with increased phosphorylation, decrease in the phosphatases activities can potentially induce hyperphosphorylation of tau [68,69,70].

### 2.2. Connection between Tau Hyperphosphorylation and Aβ

Tau protein phosphorylation has a strong connection with soluble Aβ and this is well depicted in AD pathology. Aβ plaques disrupt neuronal excitability and thereby induce axonal bloating and neurite breakage, thus decreasing spine density [71]. There are accumulating scientific evidences that implicate the role of soluble Aβ in the induction of phosphorylation of tau protein with GSK-3β identified as an important link between Aβ and tau pathologies [72,73,74,75,76,77]. As Aβ oligomers accumulate, they downstream Akt survival signaling pathways through inhibition of the phosphatidylinositol-3-kinase (PI-3K), likewise to GSK-3β activation and subsequent tau phosphorylation [39,78]. According to studies conducted in the AD brain by Jin et. al, natural Aβ dimers at sub-nanomolar concentrations can instigate tau hyperphosphorylation at AD-specific sites. They can also disrupt the organization of microtubules and invoke neuritic dystrophy [73]. Studies have also demonstrated that following soluble Aβ oligomer treatment, hippocampal rat neurons resulted in incorrect localization of tau in the dendritic spines, thereby developing synaptic dysfunction [79]. In the somatodendritic compartment, investigation of localized early changes post AD treatment resulted in missorting of endogenous tau. The regions prevalent with missorted tau had local elevation of Ca^2+^ , loss of microtubules, decreased mitochondrial density, and increased tau phosphorylation at AD-Tau specific site [80]. Lloret et al. has showed that Aβ upregulates calcineurin 1 (RCAN1) expression while the enhanced RCAN1 levels facilitate increased tau phosphorylation through two different mechanisms. Firstly, RCAN1 impedes the activity of calcineurin, which takes part in tau dephosphorylation, and secondly, RCAN1 upregulates the activity of GSK-3β. Therefore, overexpression of RCAN1 has a strong connection to AD neuropathology [81,82,83]. Porta et al. conducted studies in primary neurons that exhibited significant defiance to cell death under oxidative stress conditions that can be regressed by overexpression of RCAN1 in knockout mice [84]. Aβ42 oligomers might induce stress in the endoplasmic reticulum where the released Ca^2+^ activates GSK-3β and subsequently enhances tau phosphorylation [85]. Aβ species that are neurotoxic may bind to the cysteine-rich domain of the Wnt- binding site and thus impede the canonical Wnt pathway, thereby further modulating the activity of GSK-3β [86]. 

### 2.3. Aβ-Facilitated Increase in Tau phosphorylation in Animal Models

Animal model studies investigating AD associated tau phosphorylation is instrumental in analyzing the underlying mechanisms and possible therapy options for the disease. Inhibition of GSK-3β is crucial in interrupting tau hyperphosphorylation and subsequent AD progression [87]. Several animal models have been studied over the last decade in order to further investigate the role of GSK-3β inhibitors on Aβ-mediated increase in tau hyperphosphorylation [88]. At larval stage, injection of Aβ42 in the hindbrain ventricle of zebrafish embryos produced a decline in cognitive functions and enhanced GSK-3β site-specific tau phosphorylation. A potent GSK-3β inhibitor—Lithium Chloride was successful in reversing these specific behavioral and molecular effects [76]. Chabrier et al. has shown that double-transgenic mouse models that express low levels of arctic mutant Aβ imitates the soluble Aβ levels consequent with early AD. Soluble Aβ promote the decline of cognitive functions and also influence tau progression significantly [89]. Studies conducted on triple transgenic (3Xg-AD) mice also reaffirmed that with increased aggregation of Aβ oligomers and pathological tau forms are exist togetherr [90]. It is understood that in AD, Aβ-induced tau pathology treatment with γ-secretase modulators also attenuates phosphorylated tau levels in animal models [90,91]. Specifically, protein kinase Akt phosphorylates GSK-3β at Ser9 and thereby instigates its inhibition in physiological conditions [37,92]. In the prevention of Aβ-induced long-term potentiation (LTP) inhibition, both caspase-3 and GSK-3 inhibitors were effective, thus underlying the potential of targeting GSK-3 in the prevention of cognitive impairment in AD. These models have been useful in predicting toxicological profiles in human AD patients, however further *in vitro* and *in vivo* studies are warranted for a more robust correlation.

## 3. Tau Mediated Neurotoxicity, Secretion and Inter-Cellular Transfer

### 3.1. Neurotoxicity from Tau

Characterization of tau species accountable for AD pathogenesis and neurotoxicity is of significant interest in the field. Post-mortem studies conducted in AD patients have proven a strong correlation between the density of NFTs and respective cognitive impairments [93,94]. Pontecorvo et al. and Choi et al. have recently used tau Positron Emission Tomography (PET) tracers to conduct imaging studies involving selective tau species that mimic tau pathology and the progression of the disease as described by the Braak stages. Their findings suggested a strong, positive association between the decline of cognitive functions and tau aggregation, with implied harmful effects of insoluble tau [95,96]. Tau aggregation is further enhanced by the caspase cleavage at the C terminus of tau [97]. Caspases (cysteine aspartate proteases) belong to a group of enzyme proteases that have instrumental roles in neuroinflammation and cell death [98]. Specific caspases known as executioner caspases facilitate apoptosis and nuclear fragmentation however more recent studies have revealed that caspases are activated in the brain of individuals suffering from AD [99,100,101]. Proapoptotic proteins in the brains of patients with AD are upregulated due to the caspase activity [102]. Furthermore, caspase-cleavage of tau and subsequent NFT formation has resulted in apoptosis in neurons of the AD brain in a number of recent investigations [102,103,104,105].

In human tau transfected HEK293 cell lines, NFT disrupted cell metabolism, like proteasome activity [106]. PHF-Tau obtained from the brains suffering from AD interacted with the 20S-subunit of this proteasome, thereby inhibiting the activity [107]. NFT-mediated decrease of the activity of this proteasome led to an aberrant protein accumulation, thus initiated a network of processes, ultimately leading to the death of neurons [108]. As observed in AD, the post-synaptic localization of pathologic Tau may be attributed to neurotoxicity as well. Dendritic tau was seen to communicate with proto-oncogene tyrosine-protein kinase Fyn *in vivo*, thereby facilitating Aβ toxicity through Fyn/NMDA receptors (NR)/PSD95 coupling that are known for promoting excitotoxicity [16]. The level of native soluble tau and its physiological functions are attenuated by the pathological aggregation of Tau, thereby inducing resultant inimical effects. Therefore, loss of function of Tau results in the disruption of the network of microtubules, RNA/DNA integrity, axonal transport, cell signaling and impaired signaling of insulin the AD brain [22].

### 3.2. Tau Secretion

**Figure 2:**
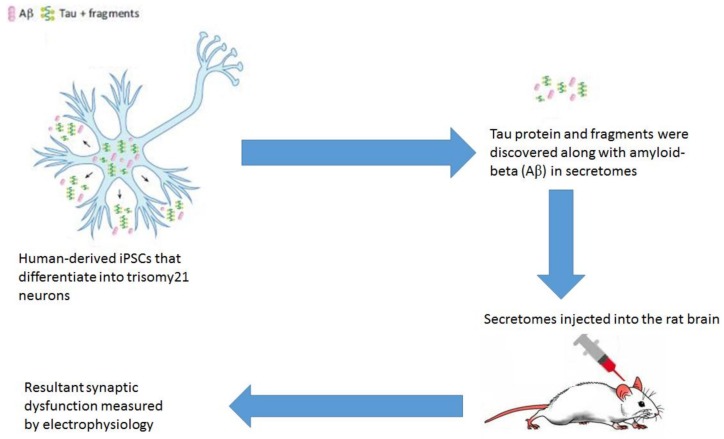
Synaptic dysfunction in rat model caused by secretomes from human derived iPSCs (Adapted from [121]).

For a long time, it was accepted that irrespective of the neurotoxicity caused by tau, the marked increase in the levels of extracellular cerebrospinal fluid (CSF)-tau was the consequence of a passive release of pathologic Tau from dead neurons in AD patients. In healthy individuals, this passive secretion of pathologic Tau generated ghost tangles, even at low levels in CSF [109]. In recent times, more captivating observations have identified Tau secretion as more of an active process [110,111]. Accordingly, in the late stages of AD, an end long decline in the levels of CSF-tau that were phosphorylated at the Thr181 site were seen to give rise to neuronal death [112]. Studies conducted in WT mice without any pre-existing neurodegeneration depicted physiological Tau secretion upon neuronal activity after the stimulation of α-amino-3-hydroxy-5-methyl-4-isoxazolepropionic acid (AMPA) receptors [113,114]. *In vitro*, it was observed that the shortening at the Asp421 site and subsequent hyper phosphorylation of tau favors its secretion [115]. Exosome-associated tau had been identified in the CSF of AD patients [116,117]. The immune system can detect extracellular Tau and subsequently initiate an antigen-driven immune response. In a study conducted in the mouse model of Tauopathy rTg4510 with mutated P301L or WT, tau prompts strong humoral immune responses followed by anti-tau antibodie [118]. Another recent interesting study related to tau secretion makes use of the observation that trisomy of chromosome 21 (trisomy-21) neuronal secretomes are able to induce synaptic dysfunction through extracellular tau [119]. In a 2018 published study, this phenomena was investigated in human induced pluripotent stem cells (iPSCs) modeling trisomy 21-related AD in murine model. Hu et al. showed that extracellular tau and related fragments with (Aβ) in secretomes of human-derived iPSCs may be obtained (these iPSCs later differentiate into trisomy21 neurons) and when these secretomes were injected into the rat brain it was seen to cause synaptic dysfunction. This secretome-induced synaptic dysfunction was measured *in vivo* by electrophysiology [120]. Figure 2 shows a schematic diagram of the process.

In healthy individuals that are prone to recognizing pathological tau, circulating tau-specific antibodies were detected that can block *in vitro* tau aggregation through the cytosolic Fc receptor TRIM21 [122,123]. Thus, it is understood that in order to obtain successful tau-immunotherapy and attenuated AD progression, identification of the most immunogenic epitopes of tau and their respective interplay with the immune system is imperative [124]. 

### 3.3. Tau Inter-Cellular Transfer

A characteristic arrangement pattern of NFT lesions in AD progression is observed during the post-mortem of AD brains where lesions begin in the transentorhinal cortex, then subsequently progressing to the hippocampus and thus affecting the temporal cortex [3,95,96,125]. This distinct progression sequence suggested a strong link between the observed clinical symptoms and relevant affected areas, thereby underlining its pivotal role in synaptic dysfunction [94,126,127]. Experimental investigation of the propagation of Tau pathology was done in transgenic P301S mice where the findings suggested the enhanced NFT accumulation of NFT in wild-type (WT) mice occurred in a time- dependent manner. In the P301S mice model of tauopathy, trans-cellular generation of tau in a prion-like state was observed [128,129]. Tau seeding was observed as an early demonstration that are present in multiple regions of the brain regions and are linked to cognitive decline and subsequent disease progression [128]. Furthermore, insoluble Tau propagated more efficiently, showing no visible signs of neurodegeneration, thus advocating that the different molecular forms of tau exist for neurotoxicity and progression [130,131]. Trans-synaptic shift of wild type dephosphorylated tau can also be depicted using a lentiviral approach [132]. Finally, another study revealed the crucial role of microglial cells in the propagation of Tau through two models of tauopathy: (1) adeno- associated virus (AAV) expressing mutated P301L tau and (2) P301S mice [133]. The findings suggested that microglial cells successfully phagocytose the aggregated tau proteins and their resulting exosomal secretion is communicable to neurons. Thus, tau propagation is inhibited by the pharmacological exhaustion of microglial cells and exosomes; underlying the instrumental functions of microglia in tau propagation and postulate it as an effective target in attenuating AD progression. 

## 4. Role of Glial Cells in AD Pathology

**Figure 3:**
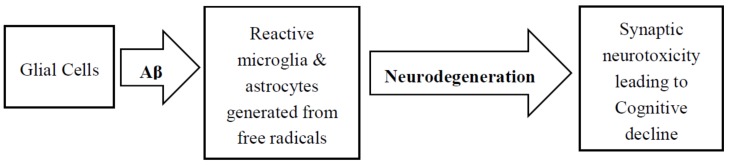
Neurodegeneration resulting from Aβ activation of glial cells.

In addition to tau and Aβ pathologies, neuroinflammatory responses involving the accumulation of reactive astrocytes and microglia very close to the amyloid deposits is another histological feature of AD. Figure 3 shows a simple scheme of neurodegeneration resulting from glial cell activation. Astrocytes supply neuronal energy in the healthy brain, participate in synaptic function, instigate synaptic pruning, and modulates neutrotrophic factor release [134,135]. Throughout neuroinflammatory instances, however, Tumor Necrosis Factor (TNFα), activated microglia-driven IL-1a, and C1q release favored the formation of reactive astrocytes known as A1. The ability to facilitate the formation of synapses and other normal functions are absent in A1 astrocytes, however, by secreting harmful factors, they induce neuronal death in the CNS [136,137]. In the AD brain, a greater proportion of A1 astrocytes were observed to produce complement protein C3, thereby asserting that this gain of toxic functions attributed to the harmful effects as there was a gradual loss of physiological properties as well [135]. In AD, morphological changes in astrocytes are instigated by neuronal tau misfolding, thereby cementing their inflammatory role through Glial fibrillary acidic protein (GFAP) regulation and subsequent secretion of pro-inflammatory factors [138,139]. In order to recreate the pathological features of astrocytic tau, transgenic mice were created that overexpressed the human tau gene [140]. Studies demonstrated that these mice develop Tau pathology in an age-dependent manner in astrocytes, and are implicated in focal neuron loss and also the disruption of the blood-brain-barrier. These phenomena further bolster the importance of reactive astrocytes in the variegated processes of different tauopathies.

Microglia has an instrumental role in AD pathology and various other tauopathies. *In-vivo* two photon imaging of the microglial cells unveil very motile and diverged procedures, thus enabling a enterprising and recurrent investigation of the healthy brain [141]. Microglia is also implicated in variegated processes including synaptic plasticity, synapse elimination or neurogenesis [142,143]. In lights of Aβ, the role of microglia on AD pathogenesis and progression was studied and reviewed extensively [144,145,146]. A complex, time-dependent effect on Aβ pathology is observed in microglial cells where they release pro-inflammatory cytokines to facilitate the removal of Aβ deposits implicated in the disease progression and overall neurotoxicity. During the disease course, longitudinal changes in the activation of microglia are measured using positron emission tomography (PET) scans. Among the patients that exhibited mild-cognitive impairment (MCI), in early stages an initial peak and another peak at a later stage of the disease were observed [147,148]. The two peaks of activation observed using PET scans might suggest a more biphasic role for microglia, however a larger cohort of patients would be required to validate this model. 

Thus, the therapeutic avenues that target microglia require a solid, thorough comprehension along with better identification and classification of the disease in individual patients. The progression of AD might be influenced by locus coeruleus (LC), which is a brain structure that generates the anti-inflammatory neurotransmitter—norepinephrine (NE) [4,149]. Its degeneration promotes a dis- inhibiting effect favoring microglial activation and facilitates the inflammatory responses [150,151]. Moreover, AD pathogenesis is further promoted by the infiltration of the brain by peripheral innate immune subsets. In AD patients with cerebral parenchyma, neutrophil infiltration was attributed to the resulting damage in cognition and amplified Tau/amyloid pathology as observed in 3xTg-AD mice [152,153]. In addition, the phenotype of APP models could potentially be influenced by the incorporation of circulating monocytes by the chemoattractant protein CCL2 along with its respective cognate receptor, CCR2. In Tg2576 APP mice, the exclusion of CCR2 increased the microglial accumulation around the blood vessels through the incorporation of mononuclear phagocytes from the bone marrow and blood, thereby promoting perivascular deposits of Aβ [154]. Studies conducted in CCR2 deficient APP/PS1 demonstrated detrimental effects on cognitive function [154,155]. Interestingly, the role of circulation monocytes in AD remains a highly debated issue since most the investigative experimental models included irradiation that compromised the blood-brain barrier [156]. Furthermore, in tau pathology, the innate immune system plays a pivotal role in the progression of the disease progression.

## 5. Diagnostic Approaches for AD Using Tau-Imaging

Gradual accumulation of tau in the brain has been widely identified as a biomarker of various neurodegenerative diseases that are collectively known as tauopathies, including AD, frontotemporal dementias (FTD), Parkinson's disease (PD) and amyolateral sclerosis (ALS) [157,158,159,160,161]. Becket et al. suggested that measurable change in tau—which is also a cerebrospinal fluid (CSF) biomarker, occurs long before the clinical symptoms of AD emerge [162]. Other studies attempted CSF testing of phosphorylated-tau/Aβ ratio for the diagnosis of Alzheimer's disease in current clinical practice, although very limited clinical uncertainties were addressed [163]. Therefore, in AD, distinctive diagnosis continues to remain elusive since the symptoms and features heavily overlap with other types of dementia. Studies conducted by Inekci et al. found serum fragments of tau exhibiting an effective role in the differential diagnosis of AD [164]. However the range of accuracy for such diagnosis is limited and has a high propensity of change with different patients. Thus, there is a strong need for a better diagnostic tool in order to identify AD in early stages. *In vivo*, selective tau imaging can potentially facilitate an improved comprehension of the aggregation of tau in the AD brain, and potentially aid in the diagnosis and treatment outcomes [165,166]. Indeed, neuropathological studies have long demonstrated a strong correlation between changes in neurodegeneration, decline in cognitive function and the deposition of tau in patients with AD [167,168,169]. Therefore, selective tau imaging would eventuate the in-vivo investigation of said communication through the measurements of changes in tau deposit levels over the course of time. 

Positron emission tomography (PET) is a noninvasive functional neuroimaging technique that allows us to design specific tracers and acquire neuronal images in small animals and humans [170]. PET imaging is suitable for examining molecular-level events, and therefore can be used for diagnosis, prognosis and recurrence of diseases. Recently, PET imaging has become very useful in current clinical practices to measure CSF Aβ42 in the brain [171,172]. In order to implement PET as an effective biomarker of tau pathology, the acquired imaging measures from PET need to be directly associated with the tau deposition in the brain. Some ideal characteristics of the PET tracers include, but not limited to, high binding affinity for paired helical filament or PHF-tau , high binding selectivity for PHF-tau, high blood–brain barrier permeability and rapid clearance from normal brain tissue [170]. The recently developed PET tracers, such as [^18^F]-AV-1451 (also known as T807), [^11^C]pyridinyl-butadienyl-benzothiazole 3 ([^11^C]PBB3) and THK-5117, are able to successfully bind to the aggregated tau in neurofibrillary tangles [173,174,175,176,177,178,179,180,181] , and can noninvasively measure the degree and extent of tau pathology in the brain. 

Two of the well-known, recently developed tau-selective PET tracers are [^18^F]T807 and [^18^F]T808 [173,182]. [^18^F]-T807 is a F-18-labeled small molecule demonstrating high selective binding and affinity to tau protein aggregates [183]. Chien et. al. demonstrated the first successful [^18^F]T807 retention of PHF-tau in the AD brain, as well as the association of [^18^F]T807 with increased disease severity [173]. Further, preliminary analyses have demonstrated that [^18^F]-T807 binding is amplified in neocortical areas of AD patients when compared with patients with normal cognitive function [184,185,186]. Moreover, [^18^F]-T807 binding at the cortical regions in AD could provide efficient diagnosis for staging of AD [187,188,189,190]. Another recent preclinical study investigating [^18^F]-T808 reported that compared to healthy controls, patients with greater probability of AD exhibited regional distinct areas of uptake in the gray matter [182]. This study concluded that compared to [^18^F]T807, [^18^F]T808 tracer exhibited more rapid tracer distribution across the brain as well as more rapid clearance from healthy brain tissue. 

Another PET tracer, [^11^C]PBB3, allows detection of tau deposits in tauopathies, including AD, progressive supranuclear palsy (PSP) and corticobasal degeneration (CBD) [175]. This particular PET tracer has been constructed with adequate radioactivity and higher quality in clinical studies, and has shown the ability to differentiate between AD patients and healthy individuals [175]. A recent study explored the associations among clinical symptoms, regional tau and Aβ deposition assessed by PET imaging with [^11^C]PBB3 and [^11^C]Pittsburgh compound-B (PiB) in AD, MCI due to AD and healthy individuals, and identified significant positive correlation between tau accumulation with Aβ pathology in all subjects, as well as significant correlation with tau burden in the healthy individuals. [191]. Further, [^11^C]PBB3 holding in the hippocampal region of AD patients verifies its binding ability to neurofibrillary tangles (NFTs). Additional investigation using PET studies with [^11^C]PBB3 tracer is required to validate its clinical usefulness in other types of tauopathies.

Other notable PET tracers include [^18^F]THK-523, [^18^F]THK-5105 and [^18^F]THK-5117 that have exhibited high binding selectivity to tau over Aβ in AD brain [192,193]. A recent study by Okamura et al. demonstrated that [^18^F]THK-5105 tracer successfully bound in sites subjected to tau deposition in the AD [176], while another study showed that [^18^F]THK-523 tracer failed to demonstrate such tau deposition in human brain [181]. Further, compared to [^18^F]THK-5105, [^18^F]THK-5117 tracer showed greater signal-to-background ratio in a PET imaging study by Shah et al. [181].These studies suggest that understanding the underlying mechanisms of tau dysregulation and incorporating them as disease-specific markers could facilitate the diagnosis of preclinical AD, and might potentially lead to therapeutic treatments. Thus, for monitoring the efficacy of anti-tau therapy in AD, selective tau imaging might be the key player with instrumental roles in diagnostic, prognostic, and progression biomarker upon clinical validation.

## 6. Immune Responses and Neuroprotection in Tau Pathology: Therapeutic Opportunities

### 6.1. Immune Responses and Neuroinflammation

Recent studies have unveiled that the incidence of Tau pathology is attributed to instigate the activation of microglia and astrocytes. Patients suffering from frontotemporal dementia (FTD) who have P301S mutation depict CD68 positive microglial cells that are activated around neurons that pertain hyperphosphorylated Tau [194]. During the regulation of cyclooxygenase-2(Cox2) and Interleukin-1β (IL1β), incidence of a strong, neuroinflammatory response was observed. During microglial activation, GFAP astrocytes that were reactive are also observed in Pick's disease [195].Thus, tau pathology facilitates the development of neuroinflammation. In various transgenic tauopathy models, neuroinflammatory changes in pathology and age-dependent microglial activation was seen in relevant CNS structures [138,196,197,198]. Activation of the innate immune response prior to the formation of hippocampal NFT implicates the involvement of soluble Tau species [198]. Recent study findings have emphasized that through the activation of inflammasome, pathological Tau could enhance the secretion of IL-1β [199]. Strategies that modulate Tau pathology impact immune response while the neuroinflammatory responses have been observed to impact Tau pathogenesis. Both Tau misfolding and neuroinflammatory response influences the impairment of behavior through loss of synaptic and neuronal integrity, henceforth facilitating the progression of pathological changes [200,201]. Microglia is involved in all the different steps occuring in the aggregation of tau, its propagation and subsequent alternation of synapse, and phosphorylation, rendering itself as an important therapeutic target in modulating AD pathogenesis and other related tauopathies. Adult neurogenesis can be induced by glial cells through the production of a potent inflammatory reaction that attenuates neuronal differentiation or progenitor proliferation [202]. Thus, existence of a hazardous loop between tau pathology, inflammation, and neurogenesis is evident and therefore, neuroprotective/therapeutic endeavors need to be carefully guided for AD attenuation.

### 6.2. Neuroprotection against AD-Tau

Whether the hyperphosphorylation of tau results in a toxic gain or systemic loss of function is of much debate in the field for a long time. Previously, it was thought that NFT and Aβ plaques resulted in the secretion of glutamate in the cortical region that led to excitotoxicity [203,204]. There is substantial evidence suggesting that the accumulation of Aβ is the primary causative process in AD [205]. The reticence of Aβ production, attenuation of soluble Aβ, and amplification of Aβ removal posit promising approaches for decreasing Aβ levels [206,207]. The strategies for best therapeutic target procurement involve inhibition of both Aβ plaque buildup, and tau hyperphosphorylation. Using non-selective γ-secretase inhibitors, potential inhibition of Aβ and subsequent decline in moderate AD were achieved in Phase III clinical trials [208,209].Ittner et al. has previously demonstrated a unique mechanism through which the phosphorylation of Threonine 205, a specific residue of tau that is of significance for protection against Aβ induced excitotoxicity [210]. The conclusions from this study suggested that phosphorylation of tau has neuroprotective functions in certain cases. The role of tau hyperphosphorylation has been extensively discussed earlier and it is imperative that the inhibition of tau hyperphosphorylation would elucidate neuroprotective effects against AD. GSK-3β hyphosphorylates tau and thus, there is a growing interest in employing GSK-3β inhibitors as neuroprotective agents [211]. Agents like valproate, neuroglobin and lithium have established efficient GSK-3β inhibition and thus showed promise for reducing AD progression [212,213,214]. A large number of kinases (e.g. Cdk5, ERK) phosphorylates tau and thus posits as potential small molecule targets in AD pathology [215]. Pedersen et al has shown high clinical efficacy in several *in vivo* AD model studies using immunotherapy against hyperphosphorylated tau [216]. Another lucrative therapy suggested through studies conducted by Kontsekova et al demonstrated effectiveness of the vaccine AAD-vac1 in targeting tau [217]. Neuroprotective approaches resulting from studies conducted with mictrobules instill the role of small molecules, which can potentially aid in the stabilization of microtubules as well as in the prevention of cytoskeletal disruption and Aβ-induced toxicity [218]. Histone deacetylase protein Sirtuin 6 (SIRT6) has been implicated in DNA repair and neurodegeneration where the lack of SIRT6 correlates with increased phosphorylation of tau [12,219].Furthermore, SIRT6 depletion in the AD brain results in increased GSK-3β activity, tau hyperphosphorylation, and subsequent neurodegeneration [220]. Thus, therapies targeting the increased expression of SIRT6 could present an effective solution towards attenuating AD. pathogenesis.

## 7. Conclusions

As AD research progresses, it is becoming evidently clear to many scientists that the role of tau in neurodegeneration is of utmost importance as we continue to solve the problems of science. The role of tau hyperphosphorylation in AD and its subsequent detrimental effects on the cognitive function and aging-related processes posit a great challenge towards the field of neuroscience. Several strategies have been implemented to combat this issue including small molecule GSK-3β inhibitors, phosphoprotein phosphatases, and tau immunotherapy. However, the efficacy of these methods yet remains to be validated in greater AD population. Tau therapies involving the immune system have also been proposed as a promising avenue against cognitive decline. Accurate diagnosis of AD remains a long-standing problem, although recent advances in tau-imaging seek to provide a potential solution. Much success has been achieved in recent times with *in vivo* PET imaging of tau and its implication in the diagnosis of early-stage AD. The polychromatic roles of tau have the propensity to amplify further beyond the current knowledge in the field as time progresses. Better diagnosis would eventually lead towards the development of efficient therapeutic targets in AD pathology. However, more time and resources are required to further understand the processes involved in the disease progression. Future studies may possibly transpire strong therapeutic targets and thereby design effective drugs to attenuate, alleviate or possibly even cure AD.

## Figures and Tables

**Table 1:** Some major enzymes and their sites of Tau phosphorylation.

Enzyme	Phosphorylation sites	Reference
PKA	Ser-195, Ser-198, Ser199, Ser-202, Ser-214, Ser-235, Ser-258, Ser-262, Ser-324, Ser-356, Ser-409, Ser-412, Ser-413, Ser422, Ser-435, Thr-205, Thr-212, Thr-217, Thr-231	[45,46,47,48,49,50]
PKB/Akt	Ser-214, Thr-212	[51]
PKC	Ser-258, Ser-293, Ser-324, Ser-352	[52]
PKN	Ser-214, Ser-258, Ser-320, Ser-352	[52]
AMPK	Ser-262, Ser-396, Ser-404, Thr-231	[53,54]
CDK5	Ser-199, Ser-202, Ser-214, Ser-235, Ser-396, Ser-404, Thr-181, Thr-205, Thr-212, Thr-217, Thr-231	[55,56]
ERK 1/2	Ser-46, Ser-199, Ser-202, Ser-235, Ser-396, Ser-404, Ser-422, Thr-50, Thr-153, Thr-181, Thr-205, Thr-212, Thr-217	[57]
GSK-3β	Ser-46, Ser-184, Ser-199, Ser-202, Ser-214, Thr-50, Thr-181, Thr-205, Thr-212, Thr-217, Thr-231	[56,58,59,60]

Abbreviations: PKA, Protein kinase A; PKB, Protein kinase B; PKC, Protein kinase C; PKN, Protein kinase N; AMPK, Adenosine monophosphate-activated kinase; CDK5, Cyclin-dependent kinase; ERK, Extracellular signal-regulated kinase; GSK-3β, Glycogen synthase kinase-3β.
